# Supplementary material for: Development of chemokine network inhibitors using combinatorial saturation mutagenesis
Source: Commun Biol. 2025 Apr 3;8:549. doi: 10.1038/s42003-025-07778-6 (PMC11969024; doi:10.1038/s42003-025-07778-6)
Supplement: Supplementary file 4 — Supplementary Data 2 [file 42003_2025_7778_MOESM4_ESM.zip › SupplementaryData3/scripts/xlogo.tar!/./script/analysis/JK/JK_HD2XC_highest_CC_minXC_logo.pdf]

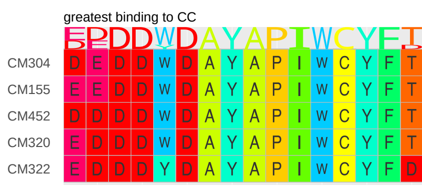

greatest binding to CC
CM304 D E D D W D A Y A P I W C Y F T
CM155 E E D D W D A Y A P I W C Y F T
CM452 D D D D W D A Y A P I W C Y F T
CM320 E D D D W D A Y A P I W C Y F T
CM322 E D D D Y D A Y A P I W C Y F D
